# Circulating tumour DNA detects somatic variants contributing to spatial and temporal intra-tumoural heterogeneity in head and neck squamous cell carcinoma

**DOI:** 10.3389/fonc.2024.1374816

**Published:** 2024-04-23

**Authors:** Karl F. B. Payne, Peter Brotherwood, Harini Suriyanarayanan, Jill M. Brooks, Nikolaos Batis, Andrew D. Beggs, Deena M. A. Gendoo, Hisham Mehanna, Paul Nankivell

**Affiliations:** ^1^ Institute of Head and Neck Studies and Education, Institute of Cancer and Genomic Sciences, University of Birmingham, Birmingham, United Kingdom; ^2^ Institute of Cancer and Genomic Sciences, University of Birmingham, Birmingham, United Kingdom; ^3^ School of Biomedical Sciences, Institute of Clinical Sciences, College of Medical and Dental Sciences, University of Birmingham, Birmingham, United Kingdom; ^4^ Institute for Interdisciplinary Data Science and AI, University of Birmingham, Birmingham, United Kingdom

**Keywords:** head and neck cancer, HNSCC (head and neck squamous cell carcinoma), liquid biopsy, Ctdna (circulating tumour DNA), intra-tumor heterogeneity, temporal heterogeneity

## Abstract

**Background:**

As circulating tumour DNA (ctDNA) liquid biopsy analysis is increasingly incorporated into modern oncological practice, establishing the impact of genomic intra-tumoural heterogeneity (ITH) upon data output is paramount. Despite advances in other cancer types the evidence base in head and neck squamous cell carcinoma (HNSCC) remains poor. We sought to investigate the utility of ctDNA to detect ITH in HNSCC.

**Methods:**

In a pilot cohort of 9 treatment-naïve HNSCC patients, DNA from two intra-tumoural sites (core and margin) was whole-exome sequenced. A 9-gene panel was designed to perform targeted sequencing on pre-treatment plasma cell-free DNA and selected post-treatment samples.

**Results:**

Rates of genomic ITH among the 9 patients was high. COSMIC variants from 19 TCGA HNSCC genes demonstrated an 86.9% heterogeneity rate (present in one tumour sub-site only). Across all patients, cell-free DNA (ctDNA) identified 12.9% (range 7.5-19.8%) of tumour-specific variants, of which 55.6% were specific to a single tumour sub-site only. CtDNA identified 79.0% (range: 55.6-90.9%) of high-frequency variants (tumour VAF>5%). Analysis of ctDNA in serial post-treatment blood samples in patients who suffered recurrence demonstrated dynamic changes in both tumour-specific and acquired variants that predicted recurrence ahead of clinical detection.

**Conclusion:**

We demonstrate that a ctDNA liquid biopsy identified spatial genomic ITH in HNSCC and reliably detected high-frequency driver mutations. Serial sampling allowed post-treatment surveillance and early identification of treatment failure.

## Introduction

Analysis of circulating tumour DNA (ctDNA) as a liquid biopsy is now regarded as a tool of modern oncology, in addition to other liquid biopsy compartments gaining traction such as circulating tumour cells (1). Evidence is strong that ctDNA can provide a means to assess tumour burden and treatment response (2, 3). Reported benefits of a liquid biopsy approach are a minimally invasive and cost effective procedure that, in contrast to a solid tissue biopsy, can be repeated serially to allow a dynamic ‘real-time’ picture of temporal heterogeneity, without the complications associated with tissue biopsy.

The clonal proliferation seen during cancer progression creates considerable intra-tumoural heterogeneity(ITH) (4). Furthermore, this evolution of the tumour genomic landscape, often driven by selection pressures, leads to the emergence of clones responsible for treatment resistance and recurrence/metastasis (R/M). Several studies have reported how ctDNA liquid biopsies can detect clonal evolution (5, 6) and be superior to a tissue biopsy to identify clinically relevant resistance mutations (7). Head and neck squamous cell carcinoma (HNSCC) is one such cancer with considerable genomic heterogeneity, both at the inter-tumoural (8) and intra-tumoural level (9–11). Despite advances in treatment, HNSCC 5-year overall survival remains largely static in the region of 50-60% (12), in part due to ITH (10) and a paucity of biomarkers to guide treatment to patient-specific therapeutic targets (13). A rising global incidence of >700,000 cases per year (12) and a shifting pattern to HPV-driven disease in a younger cohort makes this challenge all the more critical. Therefore, a liquid biopsy holds particular promise in HNSCC (14), especially given recurrence and metastasis (R/M) rates as high as 60% and 30% in HPV negative and positive disease respectively (15).

The current standard of care to assess genomic alterations driving tumour progression based upon a single site solid tissue biopsy is wholly inadequate, and is often unobtainable in cases of recurrence/metastasis (R/M). Tumour multi-region sequencing has demonstrated that considerable intra-tumoural genomic heterogeneity exists across a number of cancers (4, 16), including HNSCC (9, 17–19). Thus, single site tissue biopsies both underestimate clonal heterogeneity and are unable to assess the branched evolution occurring longitudinally and contributing to metastasis and therapeutic failure (16). Given these limitations, a liquid biopsy to assess ITH holds particular promise, especially in R/M treatment groups (20). In HNSCC, evidence continues to grow highlighting the utility of a liquid biopsy for prognostication, treatment stratification and surveillance (21–23).

The contribution of spatial heterogeneity to oncogenic variants detected within the ctDNA compartment, and the significance afforded to these, remains poorly understood. Of prime importance is the approach to ctDNA analysis - comparing a tumour-informed (specific) or tumour-naïve (agnostic) approach to oncogenic variant detection. While multiple studies have demonstrated the utility of a tumour-informed approach to monitor for recurrence and treatment response (24, 25), this may result in spatial and temporal ITH being underestimated. In contrast, a tumour naïve approach is unbiased and negates the additional workload of tumour-informed sequencing (26, 27). The primary aim of this study was to investigate if genomic spatial heterogeneity identified from multi-region sequencing of HNSCC tumours was detectable in ctDNA. Additionally, we sought to examine the efficacy of ctDNA to detect persistent or emerging clones contributing to temporal heterogeneity and potential treatment failure.

## Methods

### Sample collection and processing

**Figure 1:**
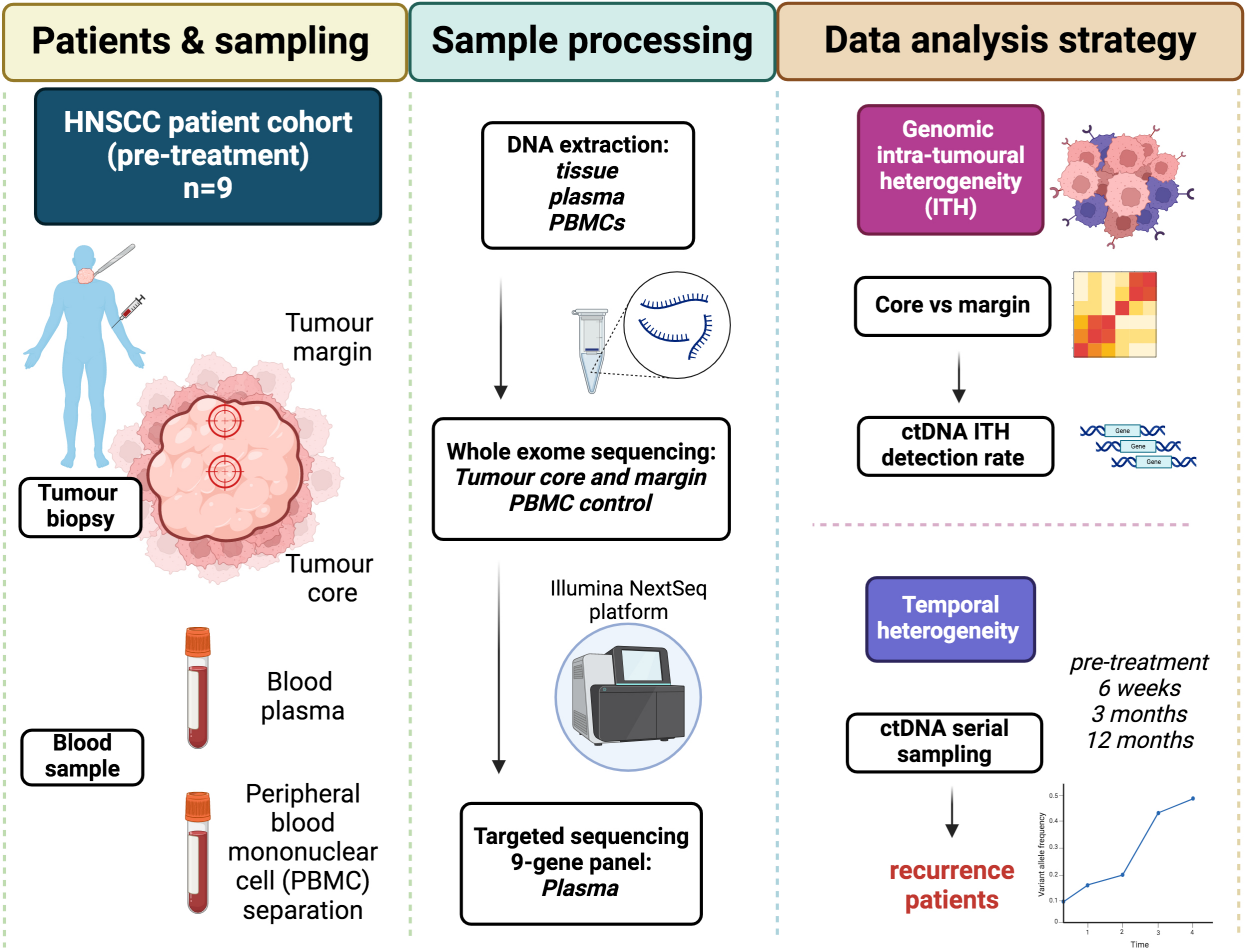
Study design – outlining patient cohort, sample collection and processing, and data analysis and outputs.

A diagrammatic representation of study design is shown in **Figure 1**. Blood and tissue samples were obtained from 9 HNSCC patients, identified from the Accelerated sample collection study (REC ref: 16/NW/0265). Tissue samples were taken at the time of resection from the core and advancing margin of the tumour. Blood samples for ctDNA analysis were taken into Streck tubes were collected at the time of resection (baseline, sample A) and then approximately 6 weeks (sample B), 3 months (sample C) and 12 months (sample D) post-treatment. Heparin blood tubes were taken at baseline to provide patient matched peripheral blood mononuclear cells (PBMCs) as per a standard lymphoprep density centrifugation protocol. PBMCs provided a negative control to remove germline and CHIP associated mutations during variant calling.

### DNA extraction

DNA was extracted from tumour tissue and PBMC samples using the DNeasy blood and tissue kit (Qiagen) and plasma DNA was extracted from 1ml of plasma using the QIAamp Circulating Nucleic Acid Kit (Qiagen) as per the manufacturers protocol.

### DNA sequencing

Tumour DNA underwent whole exome sequencing. A panel of the 9 most frequently mutated genes (*TP53, NOTCH1, PIK3CA, KMT2D, CDKN2A, CASP8, NSD1, FAT1* and *FBXW7)* observed in tumour samples was constructed for subsequent targeted plasma DNA sequencing. A deliberate decision to perform targeted sequencing of plasma samples was made to improve cost-effectiveness of the project but also demonstrate a path to clinical translational for a liquid biopsy using a pre-designed targeted panel. All sequencing was performed on the Illumina NextSeq platform at a mean read depth of 59x for tumour DNA and 84x for plasma DNA.

### Analysis of sequencing data

#### Quality control, alignment and mapping

Raw read data in FASTQ format underwent quality control using Trimmomatic v0.38 (28). Low quality bases (phred score < 20) at the 5’ and 3’ ends of each read were removed. A sliding window of 4 bases was used to remove those bases in which the average phred score of the window fell below 20. All reads shorter than 30 bp were removed. FASTQ files were aligned against human reference genome hg38 using burrows wheeler aligner (BWA) v0.7.17 (29). The resulting bam files were sorted by coordinate and indexed using Samtools v1.10 (30). Following this, duplicate reads were flagged using samblaster v0.1.26 (31).

#### Somatic variant calling

Mapped bam files for all samples were compiled into mpileup format using Samtools mpileup (30). Reads with a mapping quality above 30 were used to generate the pileup file, optical PCR duplicates were excluded. Multi-sample variant calling was implemented with VarScan v2.4.4 (32). The resulting VCF file was annotated using ANNOVAR (33) with information from COSMIC v94. Variants were called across the top 19 genes identified as highly mutated in HNSCC from TCGA data (34). Somatic variants were selected per patient by filtering out those which had an alternate allele frequency of >10% and at least 20-fold coverage in the germline control PBMC sample. Variants in this selection were initially restored if 4 or more supporting reads were detected in one or more samples. Positions which had below 20-fold coverage in the PBMC sample were restored if they had an alternate allele frequency of >20% and had 10 or more supporting reads in at least one sample. Variants which failed to meet these criteria were also restored if they were associated with a known cosmic ID related to HNSCC. Following this a variant allele frequency (VAF) threshold of 0.1% in both tumour and plasma samples was adopted. These criteria were implemented in line with increased variant calling sensitivity practices with regard recent ctDNA publications.

#### Data analysis

Plots and statistical analysis were performed using Graphpad Prism. Heterogeneity of variants between genes, patients and tumour sub-sites was compared using an unpaired t-test. Despite best efforts, we were unable to reliably perform in-depth clonality analysis of tumour tissue on our small cohort. Therefore, we assigned an arbitrary VAF of 5% to define a ‘high frequency’ variant that would infer clonality (35). The data that indicate the proportion (percentage) of somatic variants observed in tumour core and margin samples (**Figure 1**) are deposited under Github (https://github.com/DGendooLab/HNSCCgenomics/). Variants present in the primary tumour (core or margin sub-site) were labelled as ‘baseline’, while those present in post-treatment plasma DNA samples but not in the primary tumour were labelled as ‘acquired’. These labels were defined within the confines of our study design, while accepting that spatially heterogenous variants (i.e. missed by 2 site intra-tumoural sampling) may have contributed to these ‘acquired’ variants.

## Results

### Patient cohort

Demographic and clinical-pathological data for the 9 patient HNSCC cohort are displayed in **Table 1**. Mean age was 64 years (range 48-72) with two-thirds (6/9) being male. The majority of patients presented with advanced (III/IV) stage of disease, originating from an oral sub-site (8/9). Two patients suffered early treatment failure and two patients suffered recurrence following primary treatment and post-treatment samples were available.

**Table 1:** Demographic and clinical-pathological data displayed for the patient cohort.

Patient ID.	Age	Sex	T	N	Stage	Site	Primary treatment
**1**	69	F	1	2	IV	Oral	Surgery + adjuvant CRT
**2**	67	F	4	2	IV	Oral	Surgery + adjuvant CRT
**3**	72	M	4	0	IV	Oral	Surgery + adjuvant RT
**4**	61	F	2	1	III	Oropharyngeal	Primary CRT
**5**	72	M	4	0	IV	Oral	Surgery + adjuvant RT
**6**	48	M	4	2	IV	Oral	Surgery + adjuvant RT
**7**	66	M	3	0	III	Oral	Surgery
**8**	52	M	2	0	II	Oral	Surgery
**9**	70	M	1	0	I	Oral	Surgery

(CRT, chemoradiotherapy; RT, radiotherapy).

### Genomic intra-tumoural heterogeneity

We defined genomic intra-tumoural heterogeneity (ITH) as the proportion (percentage) of variants exclusive to a single tumour site (i.e. only present in core or margin sub sites but not both). Somatic variants were called from core and margin tumour site reads from the 19 most frequently mutated genes in HNSCC, according to TCGA published data (8). Variants in these genes demonstrated considerable heterogeneity (**Supplementary Figures 1A-C**) – with 96.5% of all variants being exclusive to one tumour site. The proportion of variants observed exclusively in the tumour core was 52.1%, significantly higher than seen in the tumour margin (44.4%, t-test p=0.0007).

**Figure 2:**
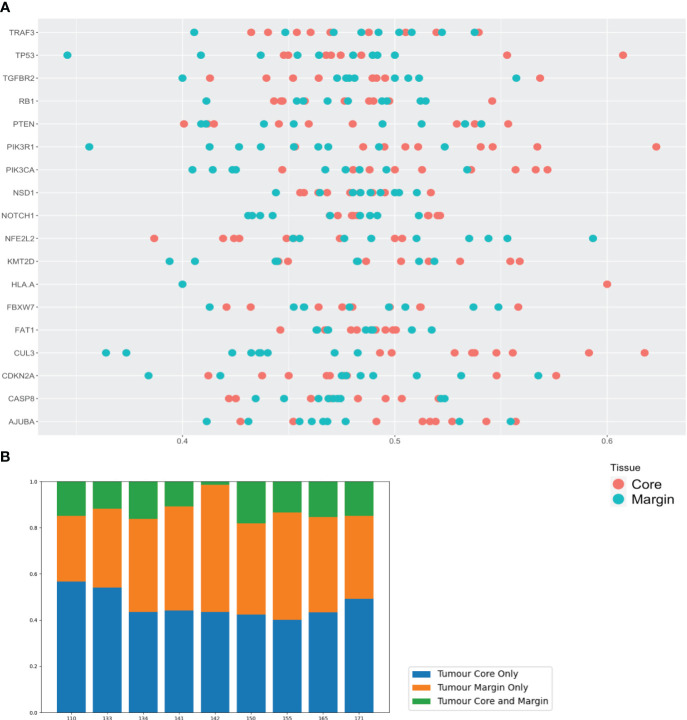
Somatic variants in tumour core and margin samples demonstrate considerable spatial genomic heterogeneity. **(A)** Linear dot plot displaying heterogeneity of COSMIC annotated gene variants from core and margin tissue sites. Key HNSCC-associated genes from TCGA data are on y-axis and heterogeneity proportion (percentage of variants exclusive to core or margin sub-site) on x-axis. Each dot the gene rows represents the mean value of all variants for a specific gene for a single patient sample. **(B)** Bar chart displaying the heterogeneity proportion (percentage) of the above variants (COSMIC annotated and TCGA HNSCC-associated) for each patient that are present in core, margin or both sub-sites.

To assign clinical relevance, COSMIC annotated variants (upper aerodigestive tract sub-set (36)) were investigated (**Figure 2A**). Differences in individual gene heterogeneity was observed, ranging from 42.9 - 100% (95% CI: 77.8-92.7%, mean 84.7%). Eight genes (*CASP8, CDKN2A, FAT1, FBXW7, KMT2D, NOTCH1, NSD1* and *TP53*) demonstrated variant ITH >90%, with <50% ITH in only one gene (*TGFBR2*)(**Supplementary Figures 1D-E**). Variants of genes *AJUBA* and *PIK3R1* were present in only 3 and 4 patient samples respectively. Certain genes appeared to demonstrate a trend for site-specific mutation - for example, *NFE2L2* and FBXW7 variants were exclusively detected at the tumour margin in 5 out of 8 patients and 3 out of 9 patients respectively. At the tumour core, *PTEN* and *RB1* demonstrated exclusivity in 3 out of 9 patients for both genes. Across all genes, the mean proportion of COSMIC annotated variants observed exclusively in the tumour core and margin was 46.3%, and 40.6% respectively (86.9% rate of heterogeneity among both sites) (**Figure 2B**).

At the individual patient level, heterogeneity of COSMIC annotated variants was consistently high at a mean 87.0% (95% CI: 86.2-93.7%, range 81.9–98.5%). There was no correlation between genomic heterogeneity and nodal metastases or advanced stage of disease (t-test, p=0.329 and 0.247 respectively).

### ctDNA identifies spatially heterogenous somatic variants

**Figure 3:**
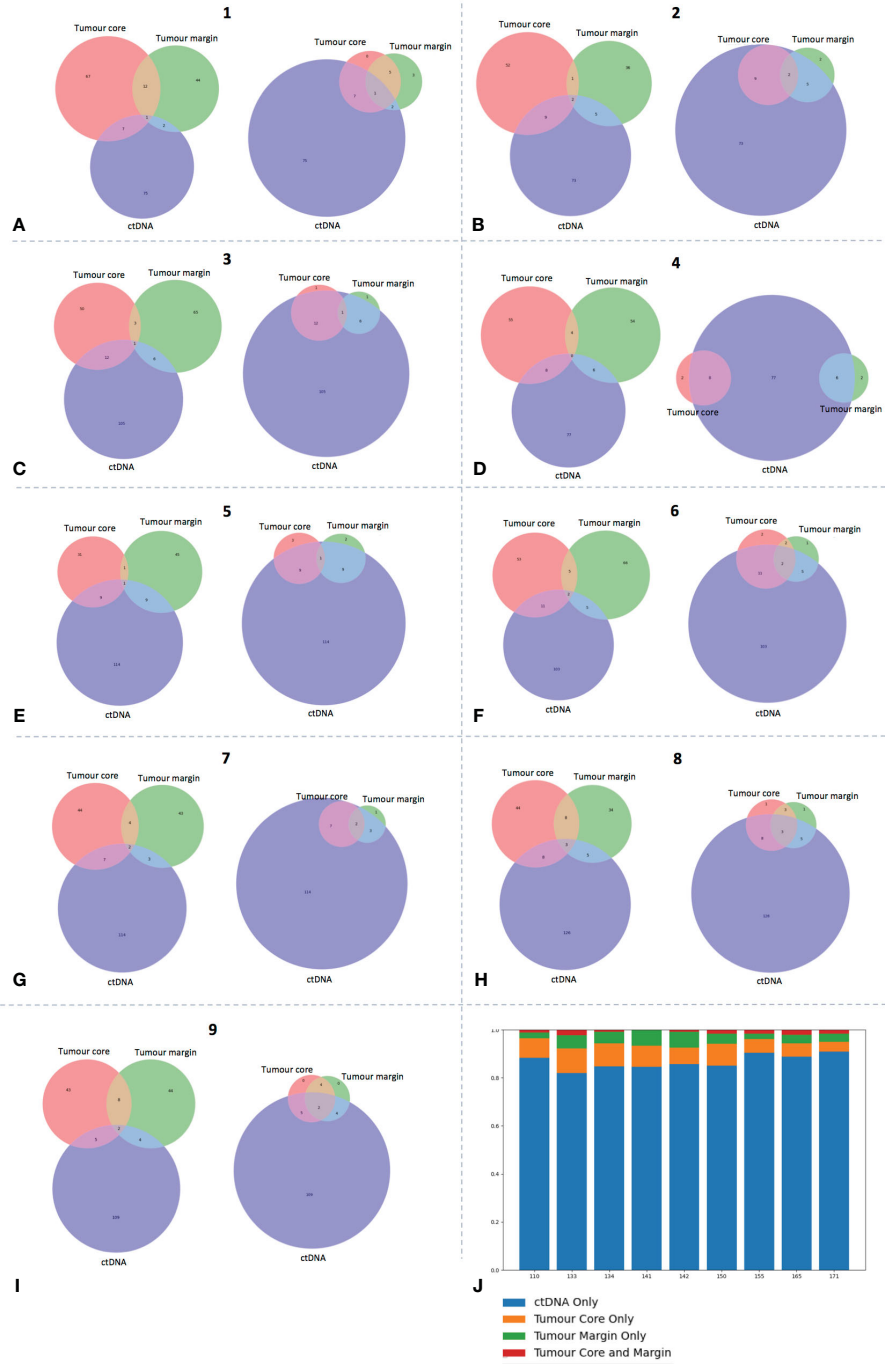
ctDNA identifies high frequency tumour-specific variants. **(A-I)** Each panel represents data from a single patient, as labelled. Venn diagrams on left for each patient demonstrate number of overlapping COSMIC annotated variants between ctDNA and core and margin tumour sites. Venn diagram on right of each panel depicts a revised VAF cut-off of 5% for tumour variant calling to demonstrate ability of ctDNA to detect these high frequency clones. **(J)** Bar chart displaying specific proportion (percentage) of *ctDNA variants* that are also in tumour sites (core, margin or both) or seen in ctDNA only.

Following the identification of genomic ITH, we sought to investigate if plasma cell-free DNA could provide a means to identify spatially heterogenous variants annotated as potential drivers in HNSCC. A hybrid approach to tumour-informed ctDNA sequencing was adopted, whereby a targeted panel of 9 genes (*TP53, NOTCH1, PIK3CA, KMT2D, CDKN2A, CASP8, NSD1, FAT1* and *FBXW7)* made up of the most frequently occurring gene variants in tumour samples was used to sequence baseline plasma cell-free DNA samples. This approach allowed detection of tumour-specific and tumour-naïve variants. Results demonstrated that cell-free DNA identified a mean of 12.9% (range 7.5-19.8%) of tumour specific variants across all patient samples (**Figure 3**). However, a high proportion of these variants were exclusive to one tumour site, demonstrating the potential of ctDNA to detect spatial ITH. In addition, a large proportion of oncogenic HNSCC variants detected in ctDNA were not seen in either tumour site (mean 86.7% across all patients, range 82.0-90.8%). Presumably, these were spatially heterogenous variants missed by tumour biopsies from only 2 sites.

Our assumption was that several of these tumour variants were either passenger or sub-clone mutations. While no variant allele frequency (VAF) cut-off is specific for clonal determination, to investigate this further, we applied a crude VAF threshold for tumour variant calling of 5%, thereby increasing the likelihood that higher frequency dominant clones were called (35) (37). Of interest, ctDNA detected a mean 79.0% (range: 55.6-90.9%) of high frequency variants across all patient samples (**Figure 3**). This figure rose to 81.9% if the outlier value from patient 110 was excluded. Of note, across all patients, 55.6% (range 39-80%) of tumour-specific COSMIC variants identified in ctDNA were in the core or margin tumour sub-site only, and therefore would have been missed with a single-site biopsy only.

### Temporal heterogeneity detected by ctDNA in post-treatment samples

We sought to investigate if longitudinal ctDNA analysis could provide a dynamic view of temporal genomic heterogeneity. Post-treatment blood samples were available for two patients (no. 1 and 6) who suffered recurrence following primary treatment. In addition to the sample taken at the time of primary surgery (day 0, sample A), blood samples were taken at approximately 6 weeks (sample B), 3 months (sample C) and 12 months (sample D) after completion of primary treatment. In those patients with recurrence, we compared variants present at baseline (i.e. innate resistance) against acquired variants that emerged following treatment, to investigate how these variants may have contributed to treatment failure. In our analysis we highlighted high frequency variants observed in ctDNA to demonstrate a picture of temporal heterogeneity – separating out those present at baseline and those acquired over time i.e. tumour-naïve.

**Figure 4:**
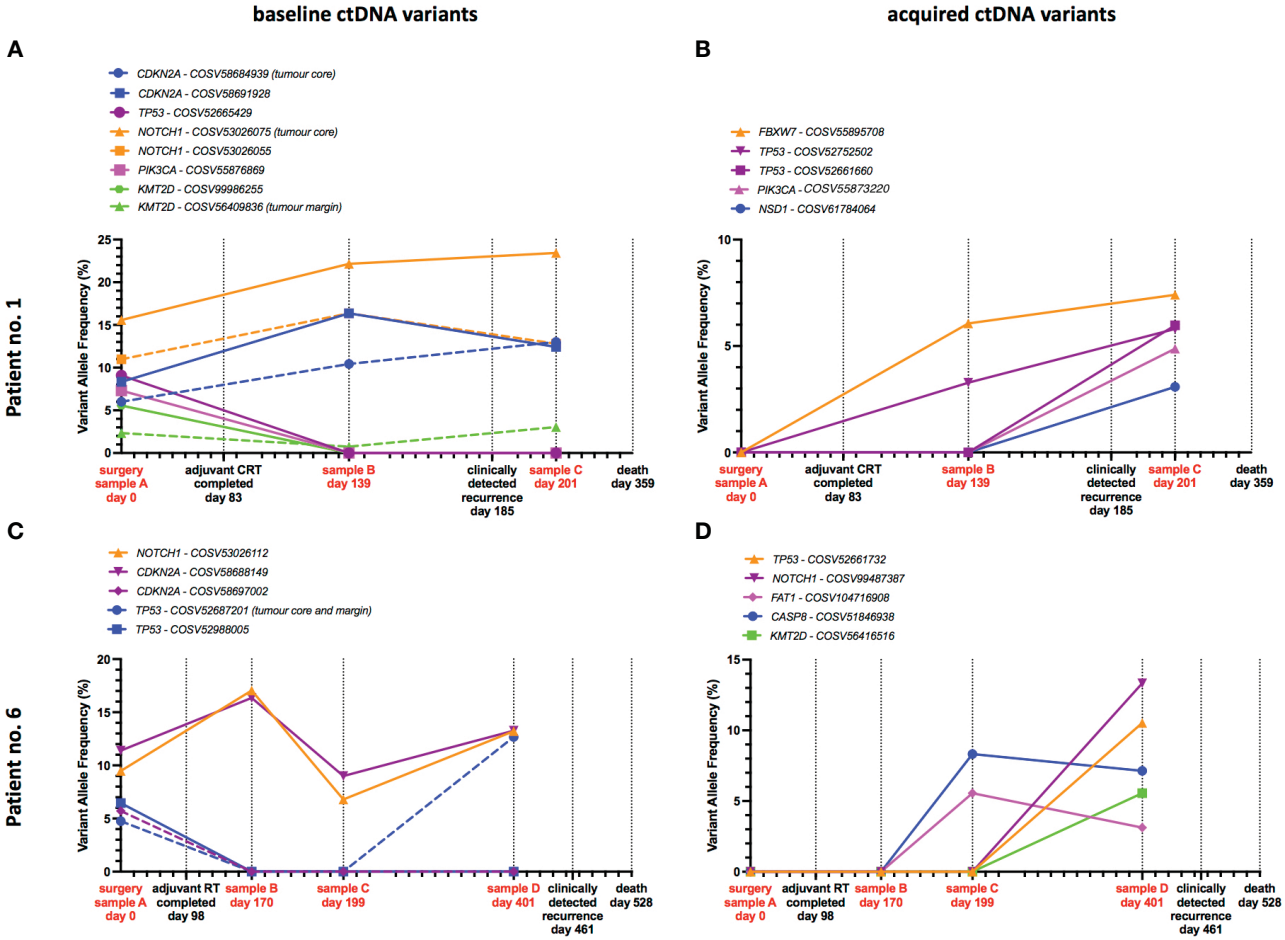
Tumour-specific and acquired variants in ctDNA predict recurrence. Timeline plots of ctDNA variant detection in two patients who suffered tumour recurrence, with ctDNA assessment at up to 3 timepoints after completion of primary treatment. Examples of variants present in ctDNA at baseline **(A, C)** and acquired post-treatment **(B, D)** are presented separately. Oncogenic variants detected in ctDNA predict tumour recurrence 46 days and 262 days before clinical detection in patients no. 1 and 6 respectively.

Clinical evidence of recurrence was observed in patient no. 1 at 102 days after completion of adjuvant CRT. In a post-treatment blood sample (sample B), taken 56 days after treatment completion, both tumour-informed and tumour-naïve variants were detected – 46 days prior to recurrence being detected clinically (**Figure 4A**). *CDKN2A* and *NOTCH1* variants present at baseline persisted at the sample B timepoint and were also present in sample C (16 days after recurrence). Tumour specific variants in *CDKN2A, NOTCH1* and *KMT2D* present at baseline were detected longitudinally in ctDNA and increased in frequency at recurrence. While *TP53, PIK3CA* and *KMT2D* variants present at baseline were not detected in sample B. Post-treatment samples also demonstrated acquired variants in *FBXW7, TP53, PIK3CA* and *NSD1* (**Figure 4B**). Two of those variants in *FBXW7* and *TP53* were present in sample B prior to clinically detected recurrence and increased in frequency in sample C – thus could have provided a means of early recurrence detection.

Patient no. 6 recurred on day 461, 363 days after completing adjuvant radiotherapy. Across 3 timepoints (samples B, C and D – sample D was taken 60 days before clinical detection of recurrence) we observed that *NOTCH1* and *CDKN2A* variants persisted from baseline and fluctuated in frequency across all timepoints (**Figure 4C**), potentially indicating minimal residual disease or early treatment failure. Of particular note was a tumour-specific *TP53* variant present at baseline, which was absent in samples B and C, but re-emerged at a high frequency in sample D – presumably a resistance clone that was dormant for a prolonged period of time (**Figure 4C**). Acquired variants of *TP53, NOTCH1, FAT1, CASP8* and *KMT2D* were also observed in samples C and D – 262 and 60 days prior to recurrence being detected (**Figure 4D**).

## Discussion

Our study has demonstrated the considerable genomic ITH present in HNSCC. As further evidence for a liquid biopsy approach, we have shown that ctDNA can reliably detect the majority of high frequency variants found in tumour tissue contributing to ITH in HNSCC. Such data is particularly novel, given the lack of previous evidence comparing tumour multi-region sequencing to ctDNA in HNSCC. Furthermore, serial ctDNA analysis identifies evidence of baseline and acquired temporal heterogeneity that may be contributing to treatment failure, thus highlighting an area of future clinical translational potential.

Several studies have utilised multi-region sequencing to quantify genomic heterogeneity across many cancers (4, 38) (39–41). A single site biopsy has been shown to miss up to 45% of spatially heterogenous tumour mutations (16), with up to 6 tumour site biopsies, or more in some cases, required to capture the majority of potentially significant oncogenic mutations (42). In HNSCC specifically, much of the historical data regarding ITH and its association with a poor clinical outcome has originated from single site biopsies, using algorithms to predict and estimate ITH (43). However, more recent studies using multi-region sequencing/sampling have demonstrated the considerable ITH in HNSCC. In one study, the detection of key driver mutations was increased from 5% to 40% when comparing single site and multi-region sampling (18), with up to 60% mutation heterogeneity when sampling 3 intra-tumour sites reported in another study (9). When sampling two intra-tumour sub-sites in our cohort we report genomic heterogeneity as >80%, further emphasising the inadequacy of single site biopsies to assess a tumours genomic architecture. In an alternative approach, Puram et al. utilised single-cell RNA seq to demonstrate the considerable phenotypic ITH in HNSCC and elucidate gene expression patterns contributing to such heterogeneity. Cells at the tumour margin were observed to be driven by an epithelial-mesenchymal-transition phenotype, which is noteworthy and may be a biological factor contributing to the genomic heterogeneity we observed at the tumour margin (versus the tumour core).

The ground breaking results from the TRACERx Lung and Renal cohorts have demonstrated the considerable genomic ITH of these cancers detectable from multi-region sequencing, while further highlighting the utility of complementary ctDNA analysis (44). While in a small pilot cohort only, we believe our study in HNSCC adds to this literature in a previously under- investigated cancer, while demonstrating similar patterns to those in the above TRACERx cancer types. Abbosh et al. described the ability of ctDNA to identify clonal *and* sub-clonal lung tumour mutations from multi-region sequencing (24). Here we report how ctDNA was able to detect >80% of high frequency tumour-specific variants in HNSCC, which are assumed to be drivers of disease. Critically, the majority of these potential drivers were heterogenous to either core or margin sub-sites, meaning that 55% (up to 80% in one case) would have been missed by a single-site biopsy only. Across all tumour-specific variants we reported a ctDNA detection rate of 12.9%. A recent systematic review reported a mean detection of 11.21% (for 7 studies with similar design) (45), which is in-keeping with our results – while they also noted differences relating to tumour site and stage. Perhaps a more notable finding from our data is the observation that a large proportion of COSMIC annotated variants observed in ctDNA were not detected in either core or margin tumour samples. Our presumption is that these variants were spatially heterogenous within the tumour outside of our 2 sampled sites. While clonality within ctDNA has been observed to be related to tumour burden and the number of metastatic lesions in which these mutations are detected (35), we were unable to infer this from our targeted dataset and small sample size. However, it has previously been demonstrated that a ‘clonality threshold’ can be defined to identify driver mutations in ctDNA with a high specificity, as per the approach adopted in our analysis (35).

The route to clinical translation still poses several unanswered questions. First and foremost is the utility of targeted sequencing or PCR mutation panels in ctDNA analysis, and their design in an empirical (tumour-naïve) versus patient-specific (tumour-informed) fashion. We demonstrated how mutations present at baseline, potentially contributing to innate tumour resistance, were able to be tracked longitudinally and give a picture of temporal heterogeneity in serial blood samples. It should be noted, we assumed that the emergence of high frequency variants in serial post-treatment ctDNA samples not seen in the primary tumour were ‘acquired’ and thus represented clonal expansion and temporal heterogeneity. However, we are unable to conclusively say that these ‘acquired’ variants were not spatially heterogenous or undetected sub-clonal mutations in the primary tumour (i.e. were missed from 2 site sampling). Within the limits of our study design we considered these as acquired post-treatment. Regardless of their origin, the profiling of these variants in a serial fashion highlighted the potential of ctDNA to provide a means to detect clinical R/M. In addition, our 2-site tumour sampling is more robust than the current clinical standard of a single site biopsy, and thus adds further emphasis on the variants that may be missed from a single tissue biopsy but can be detected serially within ctDNA. Albeit we accept that a sample of such limited patients presents hypothesis generating data only and warrants further investigation. However, previous evidence has highlighted the ability of ctDNA to track temporal clonal evolution in the R/M setting and, akin to our data, identify dynamic changes in ctDNA that predict R/M before clinical detection (24, 46), including in HNSCC (47–49). Acquired resistance mechanisms are critically important in patients suffering recurrence/metastasis (5). As in the primary setting, single site biopsies in cases of R/M underestimate the mutational load of tumours and potential mechanisms of treatment resistance. In one study, ctDNA analysis was shown to identify additional acquired resistance mutations, *not* detected in R/M biopsies, in 78% of patients (7). Using a tumour-naïve approach to define a genomic copy instability score (CNI), Schirmer et al. profiled pre- and post-treatment ctDNA samples in a larger cohort (49). While demonstrating ctDNA CNI to prognosticate for survival outcomes, they were also able to map temporal changes in CNI that predated clinical diagnosis of R/M. The utility of ctDNA to profile drivers of tumour recurrence in comparison to primary tumour mutational profiles has also been reported (47). While our data is limited in scope to address this secondary outcome, it does further stress the clinical importance of this avenue of future research. Any tumour-informed targeted ctDNA panel designed at baseline for post-treatment surveillance could miss potentially relevant genomic drivers. Hence the argument for a tumour-naïve approach is entirely valid in this regard, in addition to the cost implications of potentially unnecessary tumour sequencing, and has shown translational merit in other cancer types (27).

In this pilot study, our approach was to construct a ctDNA targeted sequencing panel comprised of the 9 most commonly mutated genes across all patients’ tumour samples, thus optimising cost-effectiveness and translational potential. We accept there is the obvious risk of missing potential driver mutations in genes not sequenced, both innate and acquired. Herein lies the debate of tumour-informed versus tumour-naïve ctDNA profiling. It was noteworthy that despite a tumour-informed approach, our panel of 9 genes strongly correlated with those found most frequently mutated in TCGA HNSCC dataset (34). What is clear from our data, and that discussed above, is that the design of targeted panels based upon single site tumour biopsies is wholly inadequate, given our increased understanding of genomic ITH, particularly in HNSCC. We believe our data adds further weight to the argument in support of tumour-naïve ctDNA profiling, as has been highlighted by other groups in HNSCC (50). Reducing the need for ‘patient-specific’ sequencing vastly increases the probability of such a test being translated to the post-treatment setting – a continued unmet clinical need in HNSCC. However, the optimum number of targeted genes required to balance cost effectiveness versus clinical utility is unknown, requiring further investigation in large cohort studies.

This study has several limitations. Designed as a pilot study, it is not possible to draw firm conclusions from this small cohort, however several trends allow hypotheses generation and pose questions for further research. Furthermore, defining VAF cut-off thresholds potentially limits the sensitivity to detect low frequency mutations – particularly in ctDNA analysis. In addition, the absence of clonality analysis on tumour and ctDNA datasets relies on the inference of driver status from VAF values and potentially limits clinical relevance of these highlighted mutations.

In summary, we present evidence for the utility of a ctDNA liquid biopsy to identify spatial genomic ITH in HNSCC. Furthermore, when performed serially in the post-treatment setting, ctDNA predicted tumour recurrence in advance of clinical detection, which succeeded in identifying dynamic changes in both tumour-specific and acquired oncogenic variants. Future research should seek to utilise ctDNA to define predictive biomarkers in HNSCC, as has been demonstrated successfully in other cancer types (51).

## Data availability statement

The datasets presented in this study can be found in online repositories. The names of the repository/repositories and accession number(s) can be found below: https://github.com/DGendooLab/HNSCCgenomics/, 01.

## Ethics statement

The studies involving humans were approved by Research ethics committee ref:16/NW/0265. The studies were conducted in accordance with the local legislation and institutional requirements. The participants provided their written informed consent to participate in this study.

## Author contributions

KP: Conceptualization, Data curation, Formal Analysis, Funding acquisition, Methodology, Writing – original draft, Writing – review & editing. PB: Data curation, Formal Analysis, Writing – original draft, Writing – review & editing. HS: Data curation, Formal Analysis, Writing – original draft, Writing – review & editing. JB: Conceptualization, Funding acquisition, Writing – original draft, Writing – review & editing. NB: Conceptualization, Writing – original draft, Writing – review & editing. AB: Data curation, Formal Analysis, Writing – original draft, Writing – review & editing. DG: Data curation, Formal Analysis, Writing – original draft, Writing – review & editing. HM: Conceptualization, Funding acquisition, Methodology, Writing – original draft, Writing – review & editing. PN: Conceptualization, Funding acquisition, Methodology, Writing – original draft, Writing – review & editing.
