# Supplementary material for: Circulating tumour DNA detects somatic variants contributing to spatial and temporal intra-tumoural heterogeneity in head and neck squamous cell carcinoma
Source: Front Oncol. 2024 Apr 23;14:1374816. doi: 10.3389/fonc.2024.1374816 (PMC11154907; doi:10.3389/fonc.2024.1374816)
Supplement: Supplementary file 1 [file DataSheet_1.pdf]

Supplementary figure 1

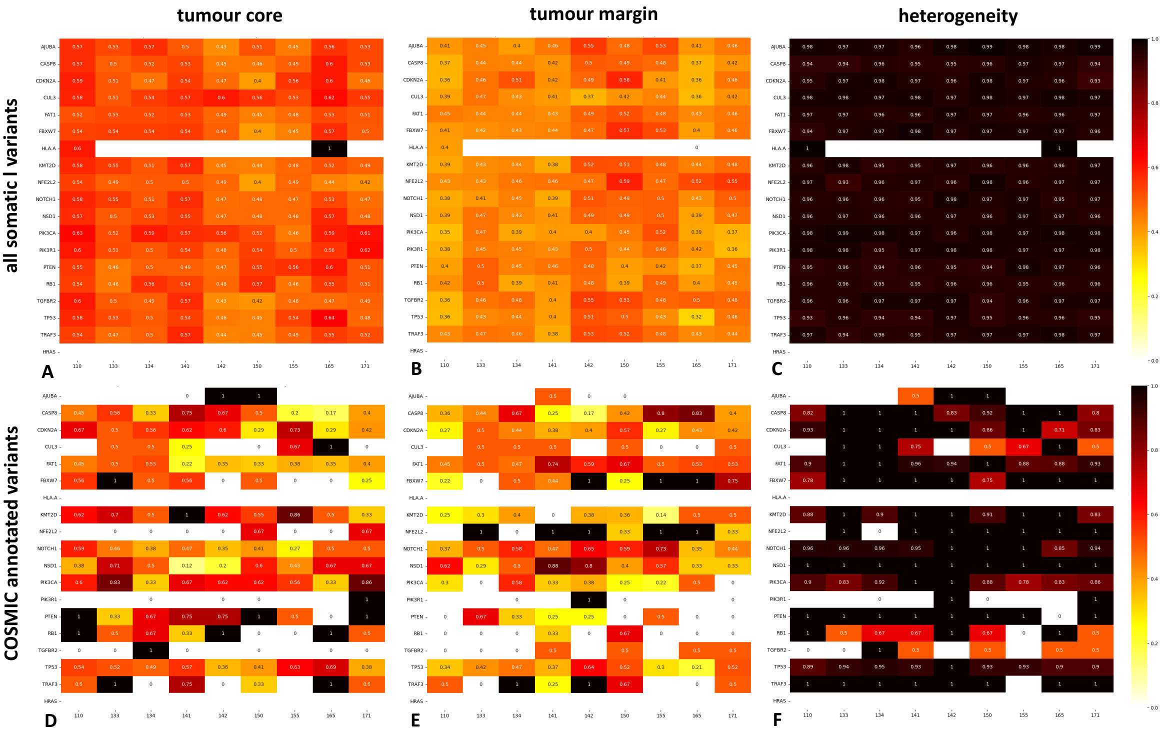

Figure 1: Somatic variants in tumour core and margin samples demonstrate considerable spatial genomic heterogeneity. Heatmaps plot key HNSCC-associated genes from TCGA data (y-axis) against patient number (x-axis). Top row (A-C) displays all somatic variants called from tumour tissue and bottom row (D-F) displays COSMIC annotated variants. Data displayed is proportion (percentage) of variants specific to that gene that are unique to a single tumour site (core or margin for each heatmap respectively). Far right column labelled 'heterogeneity' displays the proportion of variants for each gene detected in each patient that are observed exclusively to one tumour site.
